# Collaboration and followership: A stochastic model for activities in social networks

**DOI:** 10.1371/journal.pone.0223768

**Published:** 2019-10-30

**Authors:** Carolina Becatti, Irene Crimaldi, Fabio Saracco

**Affiliations:** 1 Networks Research Unit, IMT School for Advanced Studies Lucca, Lucca, Italy; 2 Axes Research Unit, IMT School for Advanced Studies Lucca, Lucca, Italy; Universitat Rovira i Virgili, SPAIN

## Abstract

In this work we investigate how future actions are influenced by the previous ones, in the specific contexts of scientific collaborations and friendships on social networks. We describe the activity of the agents, providing a model for the formation of the *bipartite network of actions and their features*. Therefore we only require to know the chronological order in which the actions are performed, and not the order in which the agents are observed. Moreover, the total number of possible features is not specified a priori but is allowed to increase along time, and new actions can independently show some new-entry features or exhibit some of the old ones. The choice of the old features is driven by a degree-fitness method: indeed, the probability that a new action shows one of the old features does not solely depend on the popularity of that feature (i.e. the number of previous actions showing it), but it is also affected by some individual traits of the agents or the features themselves, synthesized in certain quantities, called fitnesses or weights, that can have different forms and different meaning according to the specific setting considered. We show some theoretical properties of the model and provide statistical tools for the parameters’ estimation. The model has been tested on three different datasets and the numerical results are provided and discussed.

## Introduction

In the last years complex networks established as a proper tool for the description of the interactions within large systems [1–4]. The renewed attention to this field can be dated back to the well known Barabási-Albert model [5], in which the authors provide an explanation of the power-law distribution of node degrees in the World Wide Web (WWW) via a dynamic generative network model. At every step a new vertex is added and the probability to observe a new link is proportional to the number of connections (i.e. the degree) of the target node. The success of this proposal resides in the fact that only this simple rule, called preferential attachment, is able to reproduce with good accuracy the degree distribution of many real networks, such as the WWW. Even if the original mechanism was already present in the literature in a slightly different form [6, 7], the paper of Barábasi-Albert boosted the attractiveness of complex networks and other scholars delved into the investigation of the properties of generative models based on different types of dependence of the connection probabilities and the degrees [8, 9]. The preferential attachment was enriched with another ingredient, such as the fitness [10, 11]: a quantity defined per node that measures the intrinsic ability of the vertex to collect links. Then, the probability of targeting a certain node becomes the product of its fitness and degree. The effect of this new variable is to amplify or dampen the preferential attachment effect. Indeed, the presence of the fitness permits to overcome the “first move advantage” (i.e. the fact that older nodes have greater degrees by construction), thus permitting to young nodes to grow easily. Furthermore time dependence has been included by considering the possibility of node aging, i.e. multiplying the probability of link by a time dependent damping function [2, 12–14]. The importance of the previous proposals was not in the definition of the model per se, but in providing an explanation for the structure of the networks examined. For instance, the preferential attachment in [5] explains the power-law degree distribution in the World Wide Web and describes a “rich get richer” competition for links. Instead, in the fitness methods, some attributes of the nodes, not directly observed in the network, define the structure of the network (as in the case of e-mails networks, in which senders do not have access to information about the number of connection of the receivers [15]). In the same way, fitness aging [13] gives an explanation to the limited (in time) growth in citation of most of the papers.

All previous efforts were devoted to monopartite, directed or undirected, networks. A much smaller number of contributions is available for the description of the evolution of bipartite networks. In bipartite networks, nodes are divided into two different classes and only links connecting nodes belonging to different classes are allowed [1, 4]. Guillame and Latapy [16] proposed a simple model that produce a power-law degree distribution for both classes (for instance, this is the case of reviews and reviewers in the Netflix dataset). Some other dynamical models for bipartite networks were proposed for the description of specific systems. For instance, in [17] the authors propose a generative model to study the bipartite networks of lawyers and clients that develops according to a recommendation process: more popular lawyers are also more likely to be hired by new clients. Furthermore, the authors in [18] provide a framework in which the simultaneous evolution of two systems has been studied. Indeed, they analyse communities of scientists considering both the monopartite network describing the interactions among agents themselves and the bipartite semantic network in which the agents are associated to the concepts they use. Another example is [19], in which the structure of the (growing) bipartite trade network (one class includes the countries and the other one includes the exported products) was reproduced by assigning links with sequential preferential attachment, considering the degree of both nodes in the process. In order to describe the generation of an innovative product, following the idea of the “adjacent possibles” [20], new nodes (i.e. new products) are derived by the structure of an unobserved monopartite network of products describing the hierarchical productive process relations. Therefore, the evolution of the bipartite system is due to the simultaneous dynamics of an unobserved evolving network.

The present work aims at providing a generative model for the bipartite networks, where one class is formed by agents and the other one includes their actions. The starting point is the model for monopartite networks studied in [21] and its variant introduced in [22]. In [21], a set of nodes sequentially join the network, each of them showing a set of features. Each node can either exhibit new features or adopt some of the features already present in the network. This choice is regulated by a preferential attachment rule: the larger the number of nodes showing a certain feature, the greater the probability that future nodes will adopt it too. The total number of possible features is not specified a priori, but is allowed to increase along time. Differently from [16, 23], each node has been weighted with a fitness variable, that accounts for nodes’ personal ability to transmit its own features to future nodes. The model in [22] introduces some novelties in the previous context: the probability to exhibit one of the features already present in the network is defined as a mixture, i.e. a convex combination, of random choice and preferential attachment. However, neither fitnesses nor weights are introduced in the model, so that all nodes are assumed to have equal capabilities in transmitting their personal features to the newcomers. The present work moves along the same research line of the previously mentioned papers [21, 22], but with a different spirit. Indeed, the previous papers provide two different models of network formation, in which the nodes sequentially join the network and the number of common features affects the probability of connections among them. The main drawback of these two models resides in the assumed chronological order of nodes’ arrivals, which may typically be unknown (or non-relevant) in many real-world systems. In the present paper, given a system of *n* agents, we provide a model for the formation of the bipartite network of agents’ actions and their features. Therefore, this model can be applied to all settings in which agents of interest are not observed in a specific chronological order, because the assumption on the chronological order is specified on the agents’ actions only. Moreover, the probability to exhibit one of the features already observed is defined as a mixture of random choice and preferential attachment with weights, i.e. the probability of connection depends both on the features’ degrees and the fitness of the agents involved and/or of the features themselves. These weights *W*_*t*,*j*,*k*_ can have different forms and meanings according to the specific setting considered: the weight at time-step *t* of the observed feature *k* can depend on some characteristics of *k* itself, or it can be directly established by the agent performing action *t*; it may also represent the inclination of the agent performing action *t* in adopting the previous observed features, or some properties of the agent performing the previous action *j* with *k* among its features (for instance, her/his ability to transmit her/his own features).

We analyse two datasets of scientific publications (respectively IEEE for Automatic Driving, and arXiv for Theoretical High Energy Physics, or more briefly Hep-Th) and a dataset of posts of Instagram. We not only obtain a good fit of our model to the data, but our analysis also results useful in order to highlight interesting aspects of the activity of the three considered networks. Indeed, we find different variables playing a role in their evolution. In the three systems studied, we consider the degrees of the features (i.e. the popularity of, respectively, keywords in a scientific paper or hashtags on Instagram) and some fitness variables associated to the agents as drivers for the dynamics. For the scientific publications, we show a good agreement of the model to the IEEE dataset for Automatic Driving and to the arXiv dataset for Hep-Th with weights based on the number of publications or the number of co-authors of an author, the former performing better in the case of Automatic Driving. Otherwise stated, in the case of Automatic Driving the ability of an author to transmit the keywords of her/his papers, that essentially describe her/his research topics, is better reproduced by her/his number of publications, while in Hep-Th this ability is related both to the activity of the author, i.e. to the number of her/his publications, and to the number of collaborations established in her/his career. This difference can be due to the nature of the two research fields in the considered temporal window. Automatic Driving is more recent and limited, and new results drive the evolution of the research. Thus, an author transmits more keywords the more its activity in the research. Hep-Th research area, instead, is an older and structured research field, evolved in different specialized branches. In the case of Instagram, we find that the dynamics is well reproduced using the popularity of the users in a tricky sense: a standard user tends to employ many already existing hashtags, in order to acquire more visibility, while popular users mention just few already existing hashtags. Moreover, as for the previous two collaboration networks, also for the on-line social network Instagram, the relevance of an agent (with respect to the probability of transmitting her/his features) is well measured by her/his activity, that is the number of her/his actions.

The present paper is so organized. We first illustrate in detail the proposed model for the formation of the actions-features bipartite network. Then, we explain the meaning of the model parameters and the role of the weights introduced into the preferential attachment term. Some asymptotic results regarding the behavior of the total number of features and the mean number of edges in the actions-features bipartite network are collected in Section A.1 in S1 Text. The same file also contains a description of the statistical tools for the estimation of the model parameters (see Section A.2 in S1 Text). In the subsequent section we provide the general methodology used to analyse the data (the data cleaning procedure is explained in Section A.3 in S1 Text), and then we show the application of the model to the above mentioned real-world cases (IEEE, arXiv, Instagram datasets). We summarize the overall contents of the paper and recap the main findings in the last section.

## Model

Suppose to have a system of *n* agents that sequentially perform actions along time. Each agent can perform more than one action. The running of the time-steps coincide with the flow of the actions and so sometimes we use the expression “time-step *t*” in order to indicate the time of action *t*. Each action is characterized by a finite number of features and different actions can share one or more features. It is important to point out that we do not specify a priori the total number of possible features in the system, but we allow this number to increase along time. In what follows, we describe the model for the dynamical evolution of the bipartite network that collects actors’ actions on one side and the corresponding features of interest on the other side. We denote by *F* the adjacency matrix related to this network. The dynamics starts with the observation of action 1, the first action done by an agent of the considered system, that shows *N*_1_ features, where *N*_1_ is assumed Poisson distributed with parameter *α* > 0. (This distribution will be denoted from now on by the symbol Poi(*α*)). Moreover, we number the observed features with *k* from 1 to *N*_1_ and we set *F*_1,*k*_ = 1 for *k* = 1, …, *N*_1_. Then, for each consecutive action *t* ≥ 2, we have:

1. Action *t* exhibits some old features, where “old” means already shown by some of the previous actions 1, …, *t* − 1. More precisely, if *N*_*j*_ denotes the number of new features exhibited by action *j* and we set
  $$\begin{array}{cc} L_{t-1} & =\sum_{j=1}^{t-1}N_{j}\\ & =\text{the}\;\text{overall}\;\text{number}\;\text{of}\;\text{different}\;\text{observed}\;\text{features}\;\text{for}\;\text{the}\;\text{first}\;t-1\;\text{actions}, \end{array}$$ (1)
  the new action *t* can independently display each old feature *k* ∈ {1, …, *L*_*t* − 1_} with probability
  $$\begin{array}{c} P_{t}\left(k\right)=\frac{\delta }{2}+\left(1-\delta \right)\frac{\sum_{j=1}^{t-1}F_{j,k}W_{t,j,k}}{B_{t}} \end{array}$$ (2)
  where *δ* ∈ [0, 1] is a parameter, *F*_*j*,*k*_ = 1 if action *j* shows feature *k* and *F*_*j*,*k*_ = 0 otherwise, *W*_*t*,*j*,*k*_ ≥ 0 is a random weight associated to feature *k*, measured at the time of action *t* and related to the previous action *j*. Finally *B*_*t*_ is a suitable normalizing factor so that $\sum_{j=1}^{t-1}F_{j,k}W_{t,j,k}/B_{t}$ belongs to [0, 1]. We will refer to quantity (2) as the “inclusion probability” of feature *k* at time-step *t*.
2. Action *t* can also exhibit a number of new features *N*_*t*_, where *N*_*t*_ is assumed Poi(λ_*t*_)-distributed with parameter
  $$\begin{array}{c} \lambda_{t}=\frac{\alpha }{t^{1-\beta }}, \end{array}$$ (3)
  where *β* ∈ [0, 1] is a parameter. The variable *N*_*t*_ is supposed independent of *N*_1_, …, *N*_*t*−1_ and of all the appeared old features and their weights (including those of action *t*).

With the observation of the *t*^*th*^ action, all the matrix elements *F*_*t*,*k*_ with *k* ∈ {1, …, *L*_*t*_} are set equal to 1 if action *t* shows feature *k* and equal to 0 otherwise. Here is an example of a *F* matrix with *t* = 3 actions:

$$\begin{array}{c} F=(\begin{array}{ccccccccc} 1 & 1 & 1 & 1 & 0 & 0 & 0 & 0 & 0\\ 1 & 0 & 1 & 0 & 1 & 1 & 0 & 0 & 0\\ 1 & 0 & 1 & 1 & 0 & 1 & 1 & 1 & 1 \end{array}). \end{array}$$

In boldface we highlight the new features for each action: we have *N*_1_ = 4, *N*_2_ = 2, *N*_3_ = 3 and so *L*_1_ = 4, *L*_2_ = 6, *L*_3_ = 9 and, for each action *t*, we have *F*_*t*,*k*_ = **1** for each *k* ∈ {*L*_*t*−1_ + 1, …, *L*_*t*_}. Moreover, some elements *F*_*t*,*k*_, with *k* ∈ {1, …, *L*_*t*−1_}, are equal to 1 and they represent the features brought by previous actions exhibited also by action *t*.

It may be worth to note that our model resembles the one known as the “Indian buffet process” in Bayesian Statistics [24–26], but indeed there are significant differences in the definition of the inclusion probabilities: in particular, the parameter *δ* and the weights *W*_*t*,*j*,*k*_. Moreover, Bayesian Statistics deals with exchangeable sequences, while here we do not require this property. As a consequence, the role played by each parameter in (2) and (3) results more straightforward and easy to be implemented.

## Discussion of the model

We now discuss the meaning of the model parameters *α*, *β* and *δ* and the role of the weights *W*_*t*,*j*,*k*_. Some asymptotic results for the model and the statistical tools employed to estimate the model parameters are collected in Sections A.1 and A.2 in S1 Text, respectively.

### The parameters *α* and *β*

In the above model dynamics, the probability distribution of the random number *N*_*t*_ of new features brought by action *t* is regulated by the pair of parameters (*α*, *β*) (see (3)). Specifically, the larger *α*, the higher the total number of new features brought by an action, while *β* controls the asymptotic behavior of the random variable $L_{t}=\sum_{j=1}^{t}N_{j}$, i.e. the total number of features observed for the first *t* actions, as a function of *t*. In particular, it has been shown in [22] that the parameter *β* > 0 corresponds to the power-law exponent of *L*_*t*_: precisely, if *β* = 0 then the asymptotic behavior of *L*_*t*_ is logarithmic, while for *β* ∈ (0, 1] we obtain a power-law behavior with exponent *β* (see Section A.1 in S1 Text).

### The parameter *δ* and the random weights *W*_*t*,*j*,*k*_

Looking at Eq (2) of the above model dynamics, we can see that, for a generic action *t*, both the parameter *δ* and the random weights *W*_*t*,*j*,*k*_ affect the number of old features (*k* = 1, …, *L*_*t*−1_) also shown by action *t*. Specifically, the value *δ* = 1 corresponds to the pure i.i.d. case with inclusion probability equal to 1/2: an action can exhibit each feature with probability 1/2 independently of the other actions and features. The value *δ* = 0 corresponds to the case in which the inclusion probability *P*_*t*_(*k*) entirely depends on the (normalized) total weight associated to feature *k* at the time of action *t*, i.e. to the quantity

$$\begin{array}{c} \frac{\sum_{j=1}^{t-1}F_{j,k}W_{t,j,k}}{B_{t}}. \end{array}$$ (4)

In Eq (4), the term *W*_*t*,*j*,*k*_ ≥ 0 is the random weight at time-step *t* associated to feature *k* that can be related to the course of previous actions *j*. We denote this case as the “pure weighted preferential attachment case” since the larger the total weight of feature *k*, the greater the probability that also the new action will show feature *k*. When *δ* ∈ (0, 1), we have a mixture of the two cases above: the smaller *δ*, the more significant is the role played by the weighted preferential attachment in the spreading of the observed features to the new actions. In the sequel we will refer to (4) as the “weighted preferential attachment term”.

Regarding the weights, the possible ways in which they can be defined benefit of a great flexibility. Of course their meaning has to be discussed in relation to the particular application considered. For instance, the weight *W*_*t*,*j*,*k*_ can be directly assigned by the agent performing action *t* to the feature *k* in connection with the previous action *j*, or it may represent the inclination of the agent performing action *t* of adopting the previous observed features, or it may implicitly due to some properties of the agent performing the previous action *j* (for instance, her/his ability to transmit her/his own features), or even more. We here describe some general interesting frameworks:

1. If we set *W*_*t*,*j*,*k*_ = 1 for all *t*, *j*, *k* with normalizing factor *B*_*t*_ = *t*, then all the observed features have the same weight. Then the sum in the numerator of (4) becomes the popularity of feature *k*, that is the total number of previous actions that have already exhibited feature *k*, while the quantity (4) is essentially the average popularity of feature *k* (we divide by *t* instead of *t* − 1 in order to avoid the quantity (4) to be exactly equal to 1 for all the first *N*_1_ features). In this case the actions-features dynamics coincides with the nodes-features dynamics considered in [22].
2. We can assume that a positive random variable *G*_*i*_ (with *i* = 1, …, *n*) is associated to each agent in order to describe her/his ability to transmit the features of her/his actions to the others. This random variable can be seen as a static fitness as defined in [10, 11, 15]. In this case the weight *W*_*t*,*j*,*k*_ can be defined as *G*_*i*(*j*)_ (or a function of this quantity), where *i*(*j*) denotes the agent performing action *j*. In particular, we have *W*_*t*,*j*,*k*_ = *W*_*j*_, that is the weights only depend on *j*. Hence, the weight of a feature *k* is only due to the fitness of the agent that performs an action with *k* among its features and the sum in the numerator of (4) becomes the total weight of the feature *k* due to the agents that have previously exhibited it in their actions. The quantity $B_{t}=c+\sum_{h=1}^{t-1}W_{h}$ can be chosen as normalizing factor, i.e. we basically normalize by the total fitness of the agents that have performed actions 1, …, *t* − 1. Note that case 1) can be seen as a special case of the present, taking *G*_*i*_ = 1 and *c* = 1. Moreover, another interesting element to observe is that the weighted preferential attachment term (4) can be explained with an urn process. Indeed, for each feature *k*, let *t*(*k*) be the first action that has *k* as one of its features and image to have an urn with balls of two colors, say red and black, and associate an extraction from the urn to each action *t* ≥ *t*(*k*) + 1. The initial total number of balls in the urn is $c+\sum_{h=1}^{t(k)}W_{h}$, of which *W*_*t*(*k*)_ red. At each time-step *t* ≥ *t*(*k*) + 1, if the extracted ball is red then action *t* exhibits feature *k* and the composition of the urn is updated with *W*_*t*_ red balls; otherwise, action *t* does not exhibit feature *k* and the composition of the urn is updated with *W*_*t*_ black balls. Therefore quantity (4) gives the probability of extracting a red ball at time-step *t*. This is essentially the nodes-features dynamics considered in [22] with *δ* = 0 only. If we have *G*_*i*_ ≤ 1, an alternative normalizing factor is *B*_*t*_ = *t*. In this case the quantity (4) is the empirical mean of the random variables *F*_*j*,*k*_*W*_*j*_, with *j* = 1, …, *t* − 1 (again we divide by *t* instead of *t* − 1 for the same reason explained above).
3. We can extend case 2) to the case in which the fitness variables change along time and so we have *W*_*t*,*j*,*k*_ = *W*_*t*,*j*_ defined in terms of *G*_*t*,*i*(*j*)_, where *i*(*j*) denotes the agent that performs action *j* and *G*_*t*,*i*(*j*)_ is her/his fitness at the time-step of action *t*, thus following prescription similar to those of [13, 14]. We can also extend to the case in which the actions can be performed in collaboration by more than one agent. In this case the weight *W*_*t*,*j*_ can be defined as a function of the fitness at time-step *t* of all the agents performing action *j*.
4. We can set *W*_*t*,*j*,*k*_ = *W*_*t*,*k*_ for all *t*, *j*, *k* with *B*_*t*_ = *t* so that the term (4) becomes the average popularity of feature *k* adjusted by the quantity *W*_*t*,*k*_. For instance, we can take *W*_*t*,*k*_ as a decreasing function of t*(*k*) = max{*j*: 1 ≤ *j* ≤ *t* − 1 and *F*_*j*,*k*_ = 1}, which is the last action, before action *t*, that has *k* among its features. By doing so, in (4) the average popularity of *k* is discounted by the length of time between the last appearance of feature *k* and *t*. Another possibility is to use a weight *W*_*t*,*k*_ in order to give more relevance to the features already shown by the same agent performing action *t* in the previous actions. More precisely, we can denote by *i*(*j*) the agent that performs action *j* and, for each action *t*, we can define *W*_*t*,*k*_ as an increasing function of the sum ∑_*j*=1,…,*t*−1,*i*(*j*)=*i*(*t*)_ *F*_*j*,*k*_ so that the more an agent has exhibited feature *k* in her/his own previous actions, the greater the probability that also her/his new action will show feature *k*. An additional possibility is to eliminate the dependence on *t* and consider weights *W*_*t*,*j*,*k*_ = *W*_*k*_, where *W*_*k*_ can be seen as a fitness random variable associated to feature *k*.
5. We can modify case 2) by giving a different meaning to *G*_*i*_. Indeed, we can associate to each agent *i* a positive random variable *G*_*i*_ in order to describe her/his inclination of adopting the already appeared features. Then we can define the weight *W*_*t*,*j*,*k*_ as *G*_*i*(*t*)_ (or as a function of it), where *i*(*t*) denotes the agent performing action *t*. In this way, we have *W*_*t*,*j*,*k*_ = *W*_*t*_ for all *t*, *j*, *k*, that is the weights only depend on the inclination of the agent performing the action and, if we set *B*_*t*_ = *t* as in case 4), the term (4) becomes the average popularity of feature *k* adjusted by the quantity *W*_*t*_.
6. Finally, we can take *W*_*t*,*j*,*k*_ = *W*_*j*,*k*_ (i.e. depending on *j* and *k*, but not on *t*) in order to represent the weight given by the agent performing action *j* to feature *k* exhibited in this action. Therefore the total weight of feature *k* at time-step *t* is the total weight given to feature *k* by the agents who performed the previous actions.

These are just general examples of possible weights. We refer to the following applications to real datasets for special cases of the above examples. It is worth to note that the weights *W*_*t*,*j*,*k*_ may be not independent. For example, in case 5) we have exactly the same weight for all the actions performed by the same agent.

## Results

In this section we present some applications of the model to different real-world bipartite networks. In the first subsection we illustrate the general methodology used to analyse the datasets (we refer to S1 Text for the data cleaning procedure). The other subsections contain instead three examples: we first consider two different collaboration networks, the first one in the area of Automatic Driving and downloaded from the IEEE database, the second one in the research field of High Energy Physics and downloaded from the arXiv repository. In both cases, the agents are the authors, the agents’ actions are the published papers and the features are all 1-grams (nouns and adjectives) included in the title or abstract of each paper. Thus, the considered features identify the main research subjects treated in the papers. For these applications we make use of weights of the form *W*_*t*,*j*_, that are defined in terms of a fitness variable associated to the agents who performed previous action *j*, but measured at the time-step of the current action *t*. Finally, we present the last example: we study the quite popular on-line social network of Instagram, in which the users are the agents, the agents’ actions are the posted photos and, for each media, the features are the hashtags included in its description. Thus, the considered features identify the topics the considered posts refer to. For this example, we investigate two kinds of weights: weights of the form *W*_*t*_, that solely depend on some quantity related to the agent performing the current action *t*, in order to adjust the average popularity of each feature in (4), and, as in the previous two applications, weights of the form *W*_*t*,*j*_, that are defined in terms of a fitness variable associated to the agents who performed previous action *j*. In all the three applications, the weights are observable random variables. A more detailed interpretation of the considered weights is provided in each subsection.

### General methodology

For each considered applications, the analysis develops according to the same outline that we describe in the following subsections.

#### Estimation of the model parameters

We provide the estimated value of the parameters *α*, *β* and *δ* of the model by means of the tools illustrated in Section A.2 in S1 Text. For each parameter *p* ∈ {*α*, *β*, *δ*}, we also give the averaged value $\bar{p}$ of the estimates on a set of *R* realizations and the related mean squared error *MSE*(*p*). More precisely, starting from the estimated values $\hat{\alpha }$, $\hat{\beta }$ and $\hat{\delta }$ (and the observed chosen weights), we generate a sample of *R* simulated actions-features matrices and we estimate again the parameters on each realization, obtaining the values ${\hat{\alpha }}_{r}$, ${\hat{\beta }}_{r}$ and ${\hat{\delta }}_{r}$, for *r* = 1, …, *R*. We then compute, for each parameter *p* ∈ {*α*, *β*, *δ*}, the average estimate $\bar{p}$ over all the simulations and the *MSE*(*p*), as follows

$$\begin{array}{c} \bar{p}=\frac{1}{R}\sum_{r=1}^{R}{\hat{p}}_{r}\;MSE\left(p\right)=\frac{1}{R}\sum_{r=1}^{R}{\left({\hat{p}}_{r}-\hat{p}\right)}^{2}. \end{array}$$ (5)

#### Check of the asymptotic behaviors

We consider the behavior of the total number *L*_*t*_ of observed features along the time-steps *t* and we compare it with the theoretical one of the model (see Section A.1.1 in S1 Text). In particular, for each application, we verify that the power-law exponent matches the estimated parameter *β*. Moreover, we consider the behavior of the total number *e*(*t*) of edges in the real actions-features network and we compare it with the mean number *μ*_*e*_(*t*) of edges obtained averaging over *R* simulated actions-features networks, obtained by the model with the selected weights.

#### Comparison between real and simulated matrices and selection of the weights

We compare the real and simulated actions-features matrices on the basis of two groups of indicators: one regarding the spreading of the old features in the new actions, which depends on the weights, and the other one regarding the arrival process of the features, which does not depend on the weights. The first indicators allow us to select the most appropriate weights among those taken into consideration.

*Indicators for the spreading process of the old features*: We take into account the indicator

$$\begin{array}{c} {\bar{O}}_{T}=\frac{1}{\left(T-1\right)}\sum_{t=2}^{T}O_{t}\;\text{with}\;O_{t}=\sum_{k=1}^{L_{t-1}}F_{t,k}. \end{array}$$ (6)

For each action *t*, with 2 ≤ *t* ≤ *T*, the quantity *O*_*t*_ is the number of old features shown by action *t* and ${\bar{O}}_{T}$ provides the averaged value overall the set of observed actions. This indicator is computed for the real matrix, for the simulated matrix by the model with different kinds of weights, including also the constant weights equal to 1 in order to evaluate the relevance of the weights inside the dynamics. In particular, for the simulated matrices, the provided values are an average on *R* realizations, together with their sample standard deviation $\sigma_{O_{T}}$. Furthermore, in order to take into account also the not-exhibited old features (i.e. the zeros in the matrix *F*), we check also the number of correspondences, that is we compute the indicator

$$\begin{array}{c} \begin{array}{cc} & m_{O}=\frac{1}{T-1}\sum_{t=2}^{T}m_{O}\left(t\right)\;\text{with}\\ & m_{O}\left(t\right)=\frac{1}{\min(L_{t-1}^{re},L_{t-1}^{sim},k^{*})}\sum_{k=1}^{\min(L_{t-1}^{re},L_{t-1}^{sim},k^{*})}I_{\left\{F_{t,k}^{re}=F_{t,k}^{sim}\right\}}. \end{array} \end{array}$$ (7)

where we use the apex abbreviation *re* or *sim* to indicate whether the considered quantity is related to the real matrix or the simulated matrix, respectively. The meaning of the above indicator is the following. Given the simulated matrix, for a certain action *t*, the quantity *m*_*O*_(*t*) calculates the fraction of correctly attributed old features among the features in {1, …, k*} and *m*_*O*_ is the corresponding averaged values overall the set of observed actions. A value of *m*_*O*_ close to 1 indicates that a very high fraction of features has been correctly allocated by the model. We try different values of k* in order to detect the area where there are the major differences. As above, we simulate the matrix by the model with the chosen weights and with all the weights equal to 1 and the provided values are an average on *R* simulations, together with their sample standard deviations $\sigma_{m_{O}}$. On the basis of these two indicators, we select the suitable weights.

*Indicators for the arrival process of the features*: As said before, this process is not affected by the weights. We take into account the indicator

$$\begin{array}{c} {\bar{N}}_{T}=\frac{L_{T}}{T}=\frac{1}{T}\sum_{t=1}^{T}N_{t}, \end{array}$$ (8)

where *N*_*t*_ = *L*_*t*_ − *L*_*t*−1_ (with *L*_0_ = 0) is the number of new features brought by action *t* and ${\bar{N}}_{T}$ provides the averaged value overall the set of observed actions. This indicator is computed for the real matrix and for the simulated matrix. In particular, for the simulated matrix, the provided value is an average on *R* realizations, together with its sample standard deviation $\sigma_{N_{T}}$. Moreover, we consider the indicator

$$\begin{array}{c} m_{L}=\frac{1}{T-1}\sum_{t=2}^{T}m_{L}\left(t\right)\;\text{with}\;m_{L}\left(t\right)=\frac{|L_{t}^{re}-L_{t}^{sim}|}{L_{t}^{re}}, \end{array}$$ (9)

where, as above, we use the apex abbreviation *re* or *sim* to indicate whether the considered quantity is related to the real matrix or the simulated matrix, respectively. The meaning of the above indicator is the following. Given the simulated matrix, for a certain *t*, the quantity *m*_*L*_(*t*) computes the relative error committed in the total number of observed features and *m*_*L*_ is the corresponding averaged values overall the observations. A value of *m*_*L*_ close to 0 indicates that the relative error in the total number of observed features is very low. Again, the provided value is an average on *R* simulations, together with its sample standard deviation $\sigma_{m_{L}}$.

#### Predictive power of the model

Once the weights are selected, we perform a prediction analysis on the actions-features matrix: we estimate the model parameters only on a subset of the observed actions, we simulate the rest by means of the model and compare the real and simulated matrices. More precisely, fixed a time-step T* < *T*, we estimate the model parameters on the “training set” corresponding to the set of actions observed at *t* = 1, …, T*. We then employ those estimates to simulate the dynamics of the actions-features network related to the remaining set of actions at times *t* = T* + 1, …, *T*. Finally, taking the features really observed for these last actions as “test set”, we evaluate the goodness of our predictions by computing the following indicators:

$$\begin{array}{c} \begin{array}{cc} & m_{O}^{*}=\frac{1}{T-T^{*}}\sum_{t=T^{*}+1}^{T}m_{O}^{*}\left(t\right)\;\text{with}\\ & m_{O}^{*}\left(t\right)=\frac{1}{\min(L_{t-1}^{re},L_{t-1}^{sim},k^{*})}\sum_{k=1}^{\min(L_{t-1}^{re},L_{t-1}^{sim},k^{*})}I_{\left\{F_{t,k}^{re}=F_{t,k}^{sim}\right\}}\\ \text{and}\\ & m_{L}^{*}=\frac{1}{T-T^{*}}\sum_{t=T^{*}+1}^{T}m_{L}^{*}\left(t\right)\;\text{with}\;m_{L}^{*}\left(t\right)=\frac{|L_{t}^{re}-L_{t}^{sim}|}{L_{t}^{re}}, \end{array} \end{array}$$ (10)

where, as before, we use the apex abbreviation *re* or *sim* to indicate whether the considered quantity is related to the real matrix or the simulated matrix, respectively. The meaning of the above indicators is the same of *m*_*O*_ and *m*_*L*_: given a simulated matrix, for a certain action *t*, with T* + 1 ≤ *t* ≤ *T*, the quantity $m_{O}^{*}\left(t\right)$ calculates the fraction of correctly attributed old features among the features in {1, …, k*}, while $m_{L}^{*}\left(t\right)$ computes the relative error in the total number of observed features. Then, $m_{O}^{*}$ and $m_{L}^{*}$ are the corresponding averaged values over the test set of actions. Values of $m_{O}^{*}$ and $m_{L}^{*}$ respectively close to 1 and 0 indicate that, starting from the observation of the first T* actions (the training set), a very high fraction of features has been correctly predicted by the model and that the relative error in the total number of observed features is very low. The provided values are an average on *R* simulations of the model with the selected weights.

### IEEE dataset for Automatic Driving

**Fig 1:**
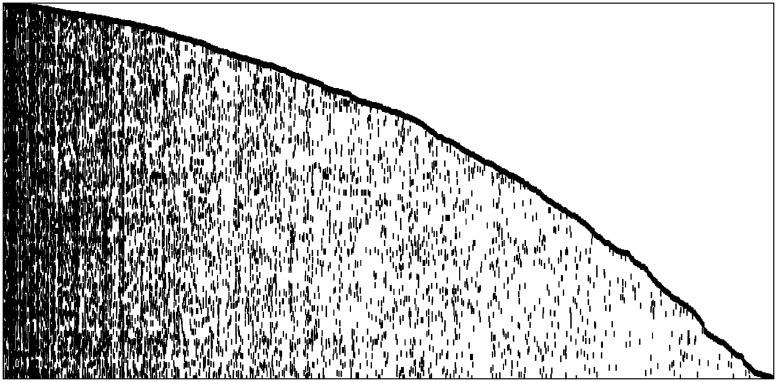
IEEE Automatic Driving dataset. Observed actions-features matrix with dimension *T* × *L*_*T*_ = 492 × 4553. Black dots represent 1 while white dots represent 0.

For the first application we have downloaded (on June 26, 2018) all papers recorded between 2000 and 2003 present in the IEEE database in the scientific research field of Automatic Driving. As in [22], we selected all papers containing at least one of the keywords: Lane Departure Warning, Lane Keeping Assist, Blindspot Detection, Rear Collision Warning, Front Distance Warning, Autonomous Emergency Braking, Pedestrian Detection, Traffic Jam Assist, Adaptive Cruise Control, Automatic Lane Change, Traffic Sign Recognition, Semi-Autonomous Parking, Remote Parking, Driver Distraction Monitor, V2V or V2I or V2X, Co-Operative Driving, Telematics & Vehicles, and Night vision. The download has yielded 492 distinct publications belonging to the required scientific field and period. For each paper we have at our disposal all the bibliographic records, such as title, full abstract, authors’ names, keywords, year of publication, date in which the paper was added to the IEEE database, and many others. The papers have been sorted chronologically according to the date in which they were added to the database. We have considered all nouns and adjectives (from now on “key-words”) included in the title or abstract as the features of the model and sorted them according to their arrival time. (See Section A.3 in S1 Text for a more detailed description of the data preparation procedure.) The features matrix obtained at the end of the cleaning procedure collects *T* = 492 papers (actions) recorded in the period 2000 − 2003 and involving *n* = 1251 distinct authors (agents) and containing *L*_*T*_ = 4553 key-words (features). The binary matrix entry *F*_*t*,*k*_ indicates whether feature *k* is present or not into the title or the abstract of the paper recorded at time-step *t*. A pictorial representation of the matrix is provided in Fig 1.

For this application, we use weights of the type 3): indeed, at each time-step *t*, we associate to each author *i* a fitness variable *G*_*t*,*i*_ that quantifies the influence of author *i* in the considered research field, and we define the weights as

$$\begin{array}{c} \begin{array}{cc} W_{t,j,k} & =W_{t,j}=e^{-1/M_{t,j}}\;\;\text{with}\;\;M_{t,j}=\max\left\{G_{t,i}:\;i\in I\left(j\right)\right\}\;\text{where}\;\\ I(j) & =\text{set}\;\text{of}\;\text{the}\;\text{agents}\;\text{performing}\;\text{action}\;j\;. \end{array} \end{array}$$ (11)

Therefore the inclusion probability in Eq (2) reads as

$$\begin{array}{c} P_{t}\left(k\right)=\frac{\delta }{2}+\left(1-\delta \right)\frac{\sum_{j=1}^{t-1}F_{j,k}\;e^{-1/M_{t,j}}}{t}. \end{array}$$ (12)

The term *M*_*t*,*j*_ is the maximum among the fitness variables *G*_*t*,*i*_ at time-step *t* of all the authors i∈I(j), i.e. the authors who published the paper appeared at time-step *j*. A high value of *G*_*t*,*i*_ should identify a person who is relevant in the considered research field so that it is likely that other scholars use the same features of her/his actions, that are the keywords related to her/his research. As a consequence, in the preferential attachment term, we give to each old feature *k* a weight that is increasing with respect to the fitness variables of the authors who included *k* in their papers. We analyse two different fitness variables:

$$\begin{array}{c} G_{t,i}^{pub}=(\text{total}\;\text{number}\;\text{of}\;\text{author}\;i’\text{s}\;\text{publications}\;\text{until}\;\text{time-step}\;t-1)+1 \end{array}$$ (13)

and

$$\begin{array}{c} G_{t,i}^{col}=(\text{total}\;\text{number}\;\text{of}\;\text{author}\;i’\text{s}\;\text{collaborators}\;\text{until}\;\text{time-step}\;t-1)+1. \end{array}$$ (14)

(Note that 1 is added in order to avoid division by zero in the previous formula (11)).

**Fig 2:**
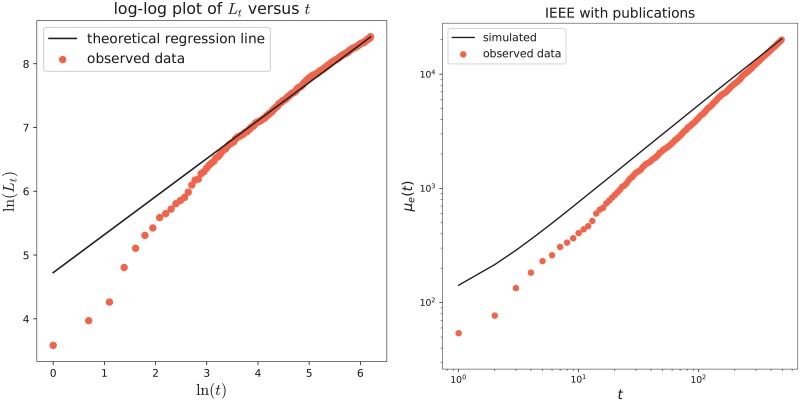
IEEE Automatic Driving dataset. Left: Plot of ln(*L*_*t*_) as a function of ln(*t*), with the power-law trend. The red dots refer to the real data and the black line gives the theoretical regression line with slope $\hat{\beta }=0.5962$. Right: Asymptotic behavior of the number of edges in the actions-features network. Red dots refer to *e*(*t*) of the real data, while the black line shows *μ*_*e*_(*t*) obtained by the model with $G_{t,i}^{pub}$ (averaging over *R* = 500 simulations).

We perform the analysis following the general methodology explained in the previous section (with *R* = 500), taking into both definition of fitness. We first estimate the model’s parameters, obtaining the results in Table 1. The estimated value for the parameter *δ*, which is zero, points out that the weighted preferential attachment term (4) plays a leading role in the inclusion probabilities. Fig 2 provides in the left panel a log-log plot of the cumulative count of new features (key-words) as a function of time (see the red dots), that clearly shows a power-law behavior. Moreover, this agrees with the theoretical property of the model stated in Section A.1.1 in S1 Text, according to which the power-law exponent has to be equal to the parameter *β* (in the figure the black line has slope equal to the estimated value for *β*, that is $\hat{\beta }=0.5962$). The goodness of fit of the model to the dataset has been evaluated through the computation of the quantities (6), (7), (8) and (9). These results are shown in Tables 2, 3 and 4. We can see that the average number of old features (i.e. the quantity ${\bar{O}}_{T}$) is well reproduced only in the case with $G_{t,i}^{pub}$, that is the case with the fitness based on the number of publications. Moreover, the average number of new features ${\bar{N}}_{T}$ perfectly matches with the real one, see Table 3. Table 4 also indicates that the model with $G_{t,i}^{pub}$ is the best performing. More precisely, for the model with the fitness $G_{t,i}^{pub}$, the computed value of ${\bar{m}}_{O}$ ranges from 88% to 97%, pointing out that a high percentage of the entries in the actions-features matrix have been correctly inferred by the model. The same value for the model with the fitness $G_{t,i}^{col}$ ranges from 83% to 96%, and, for the model with all the weights equal to 1, it ranges from 55% to 93%. The differences are more evident when we select the first k* features: indeed, with $G_{t,i}^{pub}$ we succeed to infer the value of at least 88% of the entries; while with $G_{t,i}^{col}$ and with all the weights equal to 1 the percentage remains under 88% and 70%, respectively. This means that the major difference in the performance of the different considered weights is in the first features, that are those for which the preferential attachment term (that depends on the weights) is more relevant. Note also that the actions-features matrix is more dense in the part corresponding to the first k* features. At this point, we select the model with the weights that take into account the authors’ number of publications as the best performing one for the considered dataset and in the following we focus on it. In Table 5 we evaluate the predictive power of the model: we estimate the parameters of the model only on a subset of the observed actions, respectively the 75%, 50% and 25% of the total observations; we then predict the features for the future actions {T* + 1, …, *T*} and compare the predicted and observed results by means of the indicators in (10) over the whole set of features and only on a portion of it. Finally, in the right panel of Fig 2, we provide the asymptotic behavior of the number of edges in the actions-features network: more precisely, the red dots represent the total number *e*(*t*) of edges observed in the real actions-features matrix at each time-step; while the continuous black line shows the mean number *μ*_*e*_(*t*) of edges obtained averaging over *R* = 500 simulations of the model with the chosen weights.

**Table 1:** IEEE Automatic Driving dataset. Estimation of the model parameters.

*p*	$\hat{p}$	$\bar{p}$	*MSE*(*p*)
*α*	68.533	68.589	11.765
*β*	0.5962	0.5963	0.0001
*δ* with $G_{t,i}^{pub}$	0	3.96 ⋅ 10^−5^	5.02 ⋅ 10^−9^
*δ* with $G_{t,i}^{col}$	0	4.77 ⋅ 10^−5^	7.31 ⋅ 10^−9^

**Table 2:** IEEE Automatic Driving dataset. Comparison between real and simulated actions-features matrices by means of the indicators (6).

Matrix	${\bar{O}}_{T}$	$\sigma_{O_{T}}$
Real	31.54	
Weights with $G_{t,i}^{pub}$	**31.94**	1.33
Weights with $G_{t,i}^{col}$	54.46	2.52
Weights = 1	134.39	6.66

**Table 3:** IEEE Automatic Driving dataset. Comparison between real and simulated actions-features matrices by means of the indicators (8) and (9).

Matrix	${\bar{N}}_{T}$	$\sigma_{N_{T}}$	${\bar{m}}_{L}$	$\sigma_{m_{L}}$
Real	9.25			
Simulated	9.26	0.14	0.048	0.010

**Table 4:** IEEE Automatic Driving dataset. Comparison between real and simulated actions-features matrices by means of the indicators (7), computed on the whole matrix (k* = 4553) and also taking into account only the first k* = 300, 200, 100 features.

${\bar{m}}_{O}\left(\sigma_{m_{O}}\right)$	k* = 4553	k* = 300	k* = 200	k* = 100
Weights with $G_{t,i}^{pub}$	**0.969**(0.001)	**0.914**(0.003)	**0.902**(0.003)	**0.882**(0.005)
Weights with $G_{t,i}^{col}$	0.959(0.001)	0.876(0.005)	0.855(0.007)	0.831(0.009)
Weights = 1	0.925(0.003)	0.703(0.016)	0.640(0.019)	0.548(0.028)

**Table 5:** IEEE Automatic Driving dataset. Predictions on the actions-features matrix. The indicators (10) are computed for different levels of information used as “training set”: more precisely, the different values of T* correspond to 75%, 50% and 25% of the set of the actions, respectively. Moreover, the indicator ${\bar{m}}_{O}^{*}$ is computed on the whole matrix (k* = 4553) and also taking into account only the first k* = 200 features. In the brackets, there are the sample standard deviations.

Weights with $G_{t,i}^{pub}$	${\bar{m}}_{O}^{*}\;\text{with}\;k^{*}=4553$	${\bar{m}}_{O}^{*}\;\text{with}\;k^{*}=200$	${\bar{m}}_{L}^{*}$
T* = 369	0.9857(0.0001)	0.9263(0.0012)	0.0165(0.003)
T* = 246	0.9847(0.0001)	0.9272(0.0009)	0.0600(0.006)
T* = 123	0.9828(0.0001)	0.9296(0.0008)	0.1138(0.009)

It is worth to note that the difference in the performance between the two definitions of fitness variables has a straightforward interpretation: in the considered case, i.e. for the publications in the area of Automatic Driving in the considered period, the relevance of an author (with respect to the probability of transmitting her/his features) is better measured by considering the number of her/his publications rather than the number of her/his co-authors. As we will see later on, we get a different result for the second application.

### ArXiv dataset for Theoretical High Energy Physics

**Fig 3:**
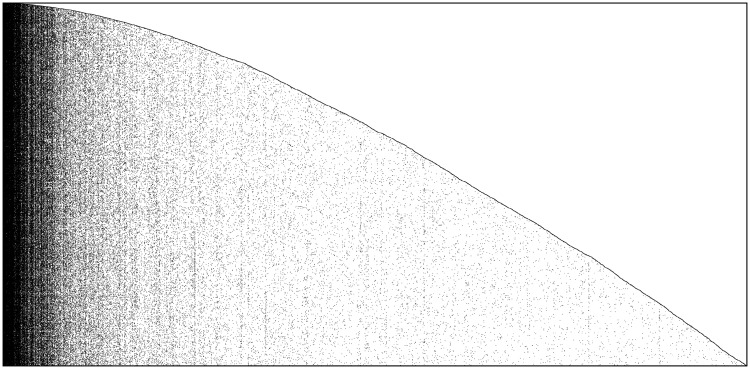
arXiv High Energy Physics dataset. Observed actions-features matrix with dimension *T* × *L*_*T*_ = 10603 × 22304. Black dots represent 1 while white dots represent 0.

The second application has been performed with the arXiv dataset of publications in the scientific area of Theoretical High Energy Physics (Hep-Th), recorded in the period 2000–2003 (the same period used for the first application), freely available from [27]. The dataset collects a sample of text files reporting the full frontispiece of each paper, so we have information on: arXiv id number, date of submission, name and email of the author who made the submission, title, authors’ names and the entire text of the abstract. From the original format we isolate the submission date and the identity number of the paper, in order to sort all papers (actions) chronologically. Then, with the final purpose of constructing the features matrix, we consider all key-words included either in the main title or in the abstract as the features of the papers and we sort them according to their time of appearance. (The complete data preparation phase is described in Section A.3 in S1 Text). We constructed the features matrix *F*, whose elements are equal to *F*_*t*,*k*_ = 1 if paper *t* includes word *k* either in the title or in the abstract and *F*_*t*,*k*_ = 0 otherwise. The result is shown in Fig 3, where the observed actions-features matrix collects *T* = 10603 papers (actions) registered between 2000 and 2003 and *L*_*T*_ = 22304 key-words appeared in the title or in the abstract (features), while the total number of involved authors (agents) is *n* = 5633.

**Fig 4:**
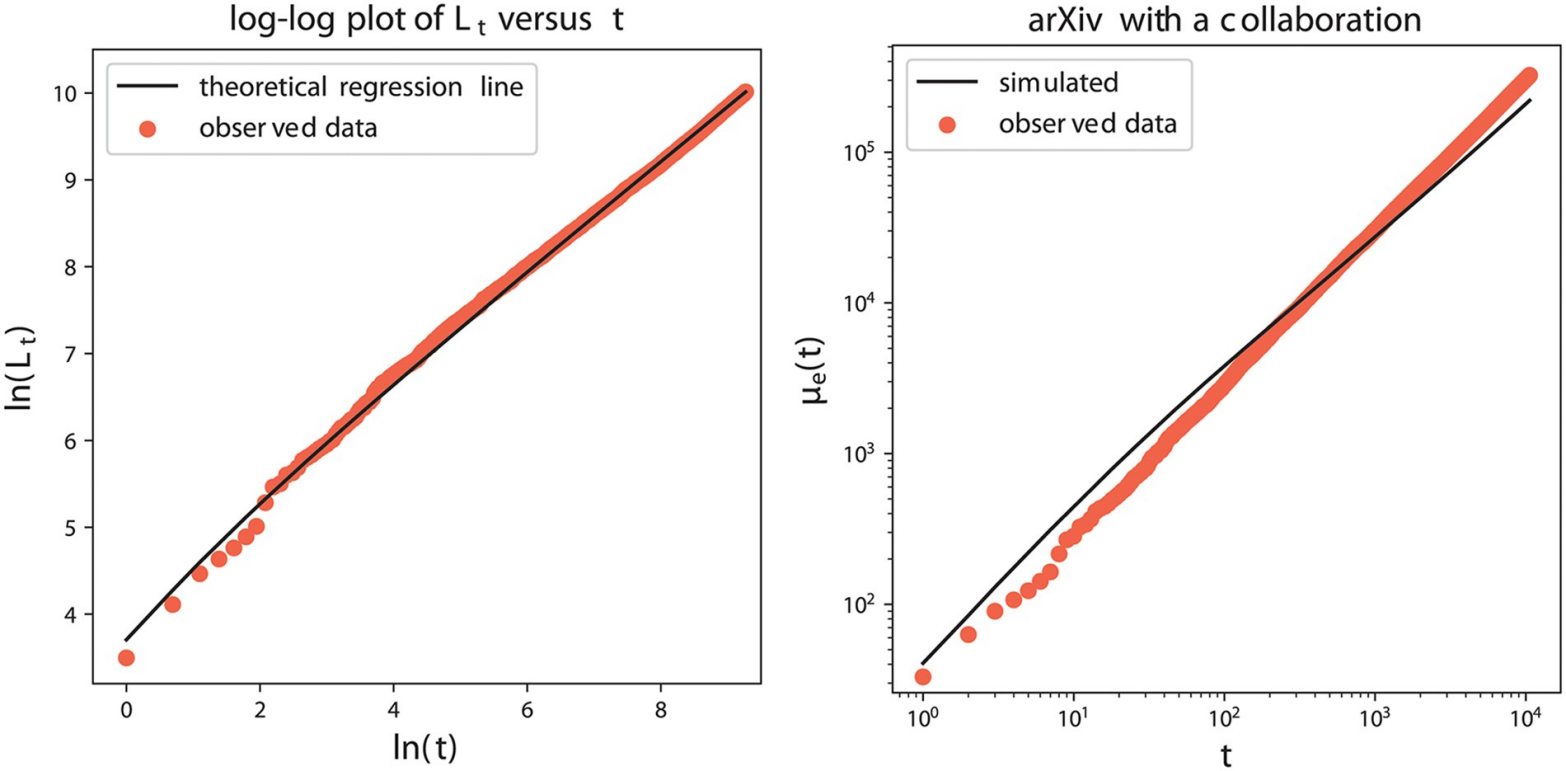
arXiv High Energy Physics dataset. Left: Plot of ln(*L*_*t*_) as a function of ln(*t*), with the power-law trend. The red dots refer to the real data and the black line gives the theoretical regression line with slope $\hat{\beta }=0.6305$. Right: Asymptotic behavior of the number of edges in the actions-features network. Red dots refer to *e*(*t*) of the real data, while the black line shows *μ*_*e*_(*t*) obtained by the model with $G_{t,i}^{col}$ (averaging over *R* = 500 simulations).

The weights for this application are defined as in the previous one, described in Eq (11). We consider again the two different definitions for the fitness term *G*_*t*,*i*_ (see (13) and (14)). The performed analysis follows the general methodology (with *R* = 500) previously explained. We first estimate the model’s parameters, obtaining the results in Table 6. Again, the estimated value for the parameter *δ* points out that the weighted preferential attachment term (4) plays a leading role in the inclusion probabilities. Fig 4 provides in the left panel a log-log plot of the cumulative count of new features (key-words) as a function of time (see the red dots), that clearly shows a power-law behavior. Moreover, this agrees with the theoretical property of the model stated in Section A.1.1 in S1 Text, according to which the power-law exponent has to be equal to the parameter *β*: indeed, in the figure the black line has slope equal to the estimated value of the parameter *β*, that is $\hat{\beta }=0.6305$. The goodness of fit of the model to the dataset has been evaluated through the computation of the quantities (6), (7), (8) and (9). These results are shown in Tables 7, 8 and 9. We can see that the model is able to perfectly reproduce the average number of new features ${\bar{N}}_{T}$. Instead, the average number of old features (i.e. the quantity ${\bar{O}}_{T}$) is under-estimated by the model with the weights based on $G_{t,i}^{pub}$ and $G_{t,i}^{col}$, while it is widely over-estimated in the case with all the weights equal to 1. The discrepancy in the values is a little smaller for the case with $G_{t,i}^{col}$ (that is the case with the fitness based on the number of collaborators). Table 8 shows that the performance of the model in reproducing the data are comparable with both the considered definitions of fitness and they are slightly better than in the case with all weights equal to one. At this point, since the best performance in reproducing ${\bar{O}}_{T}$, we select the weights that take into account the authors’ number of collaborations and the last analysis focuses on it. In Table 10 we evaluate the predictive power of the model: we estimate the parameters of the model only on a subset of the observed actions, respectively the 75%, 50% and 25% of the total observations; we then predict the features for the future actions {T* + 1, …, *T*} and compare the predicted and observed results by means of the indicators in (10) over the whole set of features and only on a portion of it. Finally, in the right panel of Fig 4, we provide the asymptotic behavior of the number of edges in the actions-features network: more precisely, the red dots represent the total number *e*(*t*) of edges observed in the real actions-features matrix at each time-step; while the continuous black line shows the mean number *μ*_*e*_(*t*) of edges obtained averaging over *R* = 500 simulations of the model with the chosen weights.

**Table 6:** arXiv High Energy Physics dataset. Estimation of the model parameters.

*p*	$\hat{p}$	$\bar{p}$	*MSE*(*p*)
*α*	40.81	40.82	2.12
*β*	0.63052	0.63054	2.05 ⋅ 10^−5^
*δ* with $G_{t,i}^{pub}$	0	1.06 ⋅ 10^−6^	3.82 ⋅ 10^−12^
*δ* with $G_{t,i}^{col}$	0	1.12 ⋅ 10^−6^	4.37 ⋅ 10^−12^

**Table 7:** arXiv High Energy Physics dataset. Comparison between real and simulated actions-features matrices by means of the indicators (6).

Matrix	${\bar{O}}_{T}$	$\sigma_{O_{T}}$
Real	28.42	
Weights with $G_{t,i}^{pub}$	15.50	0.44
Weights with $G_{t,i}^{col}$	**18.70**	0.63
Weights = 1	97.09	4.99

**Table 8:** arXiv High Energy Physics dataset. Comparison between real and simulated actions-features matrices by means of the indicators (7), computed on the whole matrix (k* = 22304) and also taking into account only the first k* = 16728, 11152, 5576 features.

${\bar{m}}_{O}\left(\sigma_{m_{O}}\right)$	k* = 22304	k* = 16728	k* = 11152	k* = 5576
Weights with $G_{t,i}^{pub}$	**0.99749**(1.8 ⋅ 10^−4^)	**0.9967**(2 ⋅ 10^−4^)	**0.9952**(3 ⋅ 10^−4^)	**0.9907**(6 ⋅ 10^−4^)
Weights with $G_{t,i}^{col}$	**0.99741**(1.8 ⋅ 10^−4^)	**0.9966**(2 ⋅ 10^−4^)	**0.9951**(4 ⋅ 10^−4^)	**0.9904**(7 ⋅ 10^−4^)
Weights = 1	0.9940(4 ⋅ 10^−4^)	0.9920(5 ⋅ 10^−4^)	0.9882(8 ⋅ 10^−4^)	0.9770(1.6 ⋅ 10^−3^)

**Table 9:** arXiv High Energy Physics dataset. Comparison between real and simulated actions-features matrices by means of the indicators (8) and (9).

Matrix	${\bar{N}}_{T}$	$\sigma_{N_{T}}$	${\bar{m}}_{L}$	$\sigma_{m_{L}}$
Real	2.10			
Simulated	2.10	0.01	0.021	0.004

**Table 10:** arXiv High Energy Physics dataset. Predictions on the actions-features matrix. The indicators (10) are computed for different levels of information used as “training set”: more precisely, the different values of T* correspond to 75%, 50% and 25% of the set of the actions, respectively. Moreover, the indicator ${\bar{m}}_{O}^{*}$ is computed on the whole matrix (k* = 22304) and also taking into account only the first k* = 11152 features. In the brackets, there are the sample standard deviations.

Weights with $G_{t,i}^{col}$	${\bar{m}}_{O}^{*}\;\text{with}\;k^{*}=22304$	${\bar{m}}_{O}^{*}\;\text{with}\;k^{*}=11152$	${\bar{m}}_{L}^{*}$
T* = 7952	0.99715(2.2 ⋅ 10^−4^)	0.99709(2.1 ⋅ 10^−4^)	0.0059(1.7 ⋅ 10^−3^)
T* = 5302	0.99709(2.2 ⋅ 10^−4^)	0.9946(4 ⋅ 10^−4^)	0.026(2 ⋅ 10^−3^)
T* = 2651	0.9947(4 ⋅ 10^−4^)	0.9948(4 ⋅ 10^−4^)	0.035(4 ⋅ 10^−3^)

Contrarily to the previous case, in this application we observe a comparable performance of the model with both the considered definitions of fitness. This means that, for the publications in High Energy Physics in the considered period, both the number of co-authors and the number of publications of an author can be considered as reasonable measures in order to evaluate her/his relevance in the research field.

### Instagram dataset

**Fig 5:**
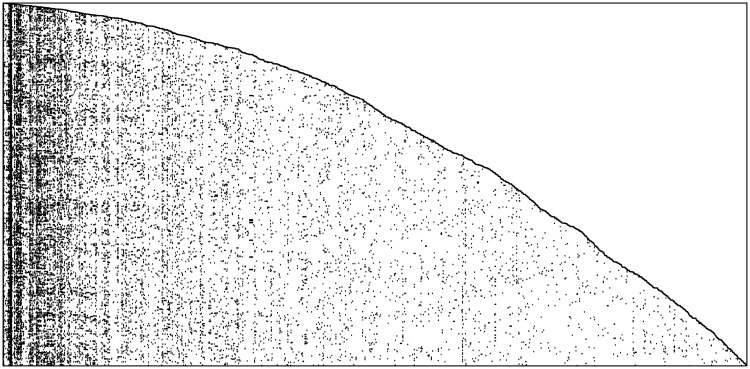
Instagram dataset. Observed actions-features matrix, with dimension *T* × *L*_*T*_ = 2151 × 5890. Black dots represent 1 while white dots represent 0.

The dataset has been crawled through the Instagram API between January 20 and February 17, 2014 and collects public media (with their author, time-stamp and set of hashtags) as well as users information (with their list of followers and followees) of a set of 2100 anonymized participants to 72 popular photographic contests that took place between October 2010 and February 2014. A detailed description of the dataset used for this application can be found in [28]. The overall media dataset records more than one million posts but, with the purpose of maximizing the density of our actions-features matrix, we considered only those posts posted during the weekends in the crawling period (Jan 20−Feb 17, 2014) in which at least 5 hashtags are used. This procedure yields a sample of *T* = 2151 posts (actions) and *L*_*T*_ = 5890 hashtags (features). The available posts were ordered chronologically according to the associate time-stamp of publication and the hashtags (features) were sorted in terms of their first appearance in a post. After this first phase of data arrangement, we constructed the actions-features matrix *F*, with *F*_*t*,*k*_ = 1 if post *t* contains hashtag *k* and *F*_*t*,*k*_ = 0 otherwise. The resulting matrix is shown in Fig 5, with non-zero values indicated by black points.

For this application, we first consider weights of the type 5), that depend on an indicator related to the underlying Instagram network. Precisely, we associate to each agent *i* the variable $G_{i}^{fol}$ defined as the number of user *i*’s followers, among those who were active during the crawling period and we set

$$\begin{array}{c} W_{t,j,k}=W_{t}=e^{-G_{i(t)}^{fol}}, \end{array}$$ (15)

where *i*(*t*) denotes the author of post *t*. Therefore the inclusion probability for hashtag *k* becomes

$$\begin{array}{c} P_{t}\left(k\right)=\frac{\delta }{2}+\left(1-\delta \right)\;\frac{\sum_{j=1}^{t-1}F_{j,k}}{t}\;e^{-G_{i(t)}^{fol}}\;, \end{array}$$ (16)

where the average popularity of hashtag *k* is exponentially discounted by the factor $G_{i(t)}^{fol}$. The decision to introduce such kind of weights was driven by the following consideration. A user with a very high number of followers identifies a person who is very popular on the social networks, an “influencer” in the extreme case. As a consequence, it may be reasonable to think that she/he is less affected by other people’s posts and, consequently, less prone to use old hashtags. For this user, the average popularity of *k* in the inclusion probability *P*_*t*_(*k*) should be less relevant. On the contrary, a user with a low number of followers may be more incline to follow the current trends and the others’ preferences and choices. It is worthwhile to point out that in the definition of the weights, we considered the number of followers of an user as fixed to the value we observed at the end of the period of observation (the crawling period). In general, it may change in time, depending on the changes in her/his network of virtual friendships. However, we assume it to be constant because of the short time span considered.

Moreover, we also use weights of the type 3), similar to those used in the previous two applications: indeed, at each time-step *t*, we associate to each user *i* the fitness variable

$$\begin{array}{c} G_{t,i}^{posts}=(\text{total}\;\text{number}\;\text{of}\;\text{user}\;i’\text{s}\;\text{posts}\;\text{until}\;\text{time-step}\;t-1)+1 \end{array}$$ (17)

and we define the weights as

$$\begin{array}{c} \begin{array}{cc} W_{t,j,k} & =W_{t,j}=e^{-1/G_{t,i(j)}^{posts}}\;\;\text{where}\;\\ & i(j)=\text{author}\;\text{of}\;\text{the}\;\text{post}\;j\;. \end{array} \end{array}$$ (18)

(As before, we add 1 in (17) in order to avoid division by zero in (18).) A high value of $G_{t,i}^{posts}$ should identify a user who is very active in the considered context so that it is likely that other users employ the same hashtags of her/his posts. Accordingly, the inclusion probability in Eq (2) reads as

$$\begin{array}{c} P_{t}\left(k\right)=\frac{\delta }{2}+\left(1-\delta \right)\frac{\sum_{j=1}^{t-1}F_{j,k}\;e^{-1/G_{t,i(j)}^{posts}}}{t}. \end{array}$$ (19)

**Fig 6:**
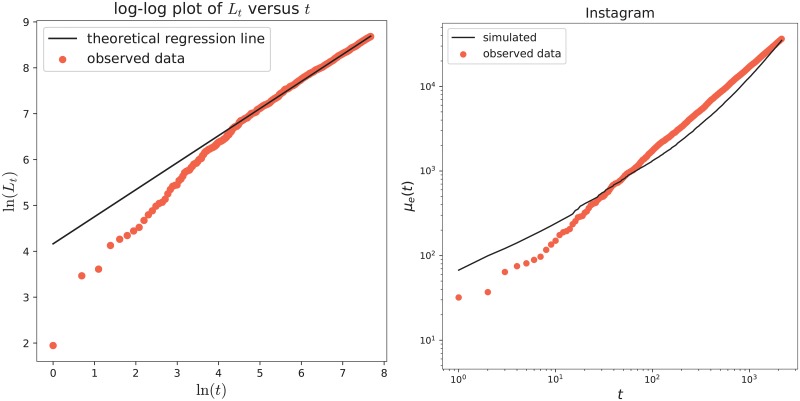
Instagram dataset. Left: Plot of ln(*L*_*t*_) as a function of ln(*t*), with the power-law trend. The red dots refer to the real data and the black line gives the theoretical regression line with slope $\hat{\beta }=0.5897$. Right: Asymptotic behavior of the number of edges in the actions-features network. Red dots refer to *e*(*t*) of the real data, while the black line shows *μ*_*e*_(*t*) obtained by the model with $G_{i}^{fol}$ (averaging over *R* = 500 simulations).

The performed analysis follows the general methodology (with *R* = 500) explained above. We first estimate the model’s parameters, obtaining the results in Table 11. Again, the estimated values for *δ* reveal that the weighted preferential attachment term (4) plays an important role in the inclusion probabilities. Fig 6 provides in the left panel a log-log plot of the cumulative count of new features (key-words) as a function of time (see the red dots), that clearly shows a power-law behavior. Moreover, this agrees with the theoretical property of the model stated in Section A.1.1 in S1 Text, according to which the power-law exponent has to be equal to the parameter *β* (in the figure the black line has slope equal to the estimated value for *β*, that is $\hat{\beta }=0.5897$). The goodness of fit of the model to the dataset has been evaluated through the computation of the quantities (6), (7), (8) and (9). These results are shown in Tables 12, 13 and 14. We can see that the average number of old features, i.e. the quantity ${\bar{O}}_{T}$, shows a good agreement with the observed quantity in the case of the model with both the considered weights, contrarily to the model with all the weights equal to one for which we obtain a much higher value. We note that the difference in the values is smaller for the case with $G_{i}^{fol}$ (i.e. the case with the fitness based on the number of followers). Moreover, the model is perfectly able to reproduce the average number of new features ${\bar{N}}_{T}$. Table 13 also indicates that the model with weights (15) (i.e. those with fitness expressed as the number of followers) shows a better performance than the one with the weights depending on the number of posts or the one with all the weights equal to one. More precisely, for the model with weights (15), the computed values of ${\bar{m}}_{O}$ ranges from 97% to 99%, pointing out that a high percentage of the entries in the actions-features matrix have been correctly inferred by the model. The differences are more evident when we select the first k* features: indeed, with with weights (15) we succeed to infer the values of at least 97% of the entries; while with weights (18) and with all the weights equal to 1 the percentage remains under 96% and 86%, respectively. This means that the major difference in the performance of the different considered weights is in the first features, that are those for which the preferential attachment term (that depend on the weights) is more relevant. Note also that the actions-features matrix is more dense in the part corresponding to the first k* features. At this point, we select the weights (15) (i.e. those taking into account the users’ number of followers) and the last analysis focuses on it. In Table 15 we evaluate the predictive power of the model with the chosen weights: we estimate the parameters of the model only on a subset of the observed actions, respectively the 75%, 50% and 25% of the total observations; we then predict the features for the future actions {T* + 1, …, *T*} and compare the predicted and observed results by means of the indicators in (10) over the whole set of features and only on a portion of it. Finally, in the right panel of Fig 6, we provide the asymptotic behavior of the number of edges in the actions-features network: more precisely, the red dots represent the total number *e*(*t*) of edges observed in the real actions-features matrix at each time-step; while the continuous black line shows the mean number *μ*_*e*_(*t*) of edges obtained averaging over *R* = 500 simulations of the model with the chosen weights.

**Table 11:** Instagram dataset: Estimation of the model parameters.

*p*	$\hat{p}$	$\bar{p}$	*MSE*(*p*)
*α*	37.896	37.901	4.086
*β*	0.5897	0.5899	7.45 ⋅ 10^−5^
*δ* with $G_{i}^{fol}$	0.0063	0.0062	2.69 ⋅ 10^−8^
*δ* with $G_{t,i}^{posts}$	0	9.80 ⋅ 10^−6^	2.93 ⋅ 10^−10^

**Table 12:** Instagram dataset. Comparison between real and simulated actions-features matrices by means of the indicators (6).

Matrix	${\bar{O}}_{T}$	$\sigma_{O_{T}}$
Real	14.23	
Weights with $G_{i}^{fol}$	**13.58**	0.21
Weights with $G_{t,i}^{posts}$	16.26	0.71
Weights = 1	79.44	4.79

**Table 13:** Instagram dataset. Comparison between real and simulated actions-features matrices by means of the indicators (7), computed on the whole matrix (k* = 5890) and also taking into account only the first k* = 500, 250, 100 features.

${\bar{m}}_{O}\left(\sigma_{m_{O}}\right)$	k* = 5890	k* = 500	k* = 250	k* = 100
Weights with $G_{i}^{fol}$	**0.9912**(0.0001)	**0.9797**(0.0001)	**0.9754**(0.0001)	**0.9666**(0.0002)
Weights with $G_{t,i}^{posts}$	0.9879(0.0003)	0.9644(0.0011)	0.9524(0.0021)	0.9327(0.0036)
Weights = 1	0.9652(0.0020)	0.8580(0.0089)	0.7720(0.0168)	0.6282(0.0291)

**Table 14:** Instagram dataset. Comparison between real and simulated actions-features matrices by means of the indicators (8) and (9).

Matrix	${\bar{N}}_{T}$	$\sigma_{N_{T}}$	${\bar{m}}_{L}$	$\sigma_{m_{L}}$
Real	2.74			
Simulated	2.74	0.03	0.0376	0.0105

**Table 15:** Instagram dataset. Predictions on the actions-features matrix. The indicators (10) are computed for different levels of information used as “training set”: more precisely, the different values of T* correspond to 75%, 50% and 25% of the set of the actions, respectively. Moreover, the indicator ${\bar{m}}_{O}^{*}$ is computed on the whole matrix (k* = 5890) and also taking into account only the first k* = 250 features. In the brackets, there are the sample standard deviations.

Weights with $G_{i}^{fol}$	${\bar{m}}_{O}^{*}\;\text{with}\;k^{*}=5890$	${\bar{m}}_{O}^{*}\;\text{with}\;k^{*}=250$	${\bar{m}}_{L}^{*}$
T* = 1613	0.99354(0.00003)	0.9775(0.0002)	0.0060(0.0024)
T* = 1076	0.99241(0.00003)	0.9754(0.0001)	0.0312(0.0055)
T* = 538	0.99019(0.00003)	0.9733(0.0001)	0.0983(0.0082)

We can conclude that for the considered dataset of Instagram, the number of followers of a user is a good measure in order to evaluate her/his inclination to employ old hashtags, but in the inverse sense: the bigger the popularity, the smaller the tendency of re-use already existing hashtags. The difference in the performance of the model with weights based on the number of followers and with weights based on the number of posts is not so significant. Therefore, as in the previous two applications, we can affirm that the relevance of an agent (with respect to the probability of transmitting her/his features) is well measured by the number of her/his actions.

### Summary of the results

We here summarize the major findings of the three considered applications.

In all the three cases we chose the weights depending on a fitness variable. In the first two applications (IEEE and arXiv), the fitness variable measures the ability of the agents (authors) to transmit the features (keywords) of their actions (publications). In the third application (Instragram) we considered two kinds of fitness: one quantifies the inclination of the agents (users) to follow the features (hashtags) of the previous actions (posts) and the other, as before, measures the ability of the agents to transmit the features of their actions. From the performed analyses of the actions-features bipartite networks, we get the following main common issues for the three applications:

- In the inclusion probabilities defined by (2), the preferential attachment term plays a relevant role, because of the small estimated values obtained for the parameter *δ*.
- The considered indicators and the plots regarding the behavior along time of the total number of observed features *L*_*t*_ show a good fit between the model with the selected weights and the real datasets. In particular, the power-law behavior of *L*_*t*_ perfectly matches the theoretical one with the estimated parameter $\hat{\beta }$ as the power-law exponent, and a high percentage of the entries of the actions-features matrix is successfully inferred with the model. Moreover, a good performance is also obtained when making a prediction analysis, i.e. testing the percentage of the entries that are successfully recovered by the model providing it with different levels of information. Regarding the plot of *μ*_*e*_(*t*), we note that the observed total number of edges is well replicated in all applications. Nevertheless, the plots of its dynamics show that the slope of the simulated curve and the real one asymptotically match only in the case of IEEE dataset. In the other applications the two curve intersect, but their slope are not the same. The different performance in replicating the dynamics of *L*_*t*_ and *μ*_*e*_(*t*) reveal that the arrival process of the features is simpler and well captured by the proposed model (recall that in the model the dynamics of *L*_*t*_ does not depend on the weights, but only on the parameters *α* and *β*) than the selection mechanism of the old features. Therefore, it is hard to recover the whole dynamics of *μ*_*e*_(*t*) along time. However, as said before, in all the considered applications, the proposed dynamics for the selection of old features (that is the inclusion probabilities endowed with suitable observed random weights and a single estimated parameter *δ*) show a good performance with respect to the employed indicators.
- With respect to the “flat weights”, i.e. all weights equal to 1, the selected weights guarantee a better agreement with the real actions-features matrices. The difference in the performance of the model with different weights is put in evidence by the indicator ${\bar{O}}_{T}$ and it is also evident when we consider a subset of the overall set of the observed features for the computation of the indicator ${\bar{m}}_{O}$. Indeed, the actions-features matrices appear more dense in the part corresponding to the first features and these features are those for which the preferential attachment term, that depends on the weights, is more relevant.

## Conclusions

In this work we have presented our contribution to the stream of literature regarding stochastic models for networks formation. With respect to the previous publications, the present paper introduces some novelties. First of all, our focus is to define a model for the bipartite network that describes the activity of some agents, studying the behavior in time of agents’ actions and the features shown by these actions. Therefore, we only assume to know the chronological order in which we observe the agents’ actions, and not the order in which the agents arrive. Second, we extend the concept of “preferential attachment with weights” [10, 11] to this framework. The weights can have different forms and meanings according to the specific setting considered and play an important role since the probability that a future action shows a certain feature depends, not only on its popularity (i.e. the number of previous actions showing the feature) as stated by the preferential attachment rule, but also on some characteristics of the agents and/or the features themselves. For instance, the weights may give information regarding the ability of an agent to transmit the features of her/his actions to the future actions, or the inclination of an agent to adopt the features shown in the past.

Summarizing, we first provide a full description of the model dynamics and interpretation of the included parameters and variables, also showing some theoretical results regarding the asymptotic properties of some important quantities. Moreover, we illustrate the necessary tools in order to estimate the parameters of the model and we consider three different applications. For each of them, we evaluate the goodness of fit of the model to the data by checking the theoretical asymptotic properties of the model in the real data, by comparing several indicators computed both on the real and simulated matrices, as well as testing the ability of the model as a predictive instrument in order to forecast which features will be shown by future actions. All in all, the analyses point out a good fit of the model and a good performance of the adopted tools in all the three considered cases.

The model and the related analysis have been able to detect some interesting aspects that characterize the different examined contexts. In the first two applications (IEEE and arXiv) we examined the publications in the scientific areas of Automatic Driving and of High Energy Physics (briefly Hep-Th) and we took into account two kinds of fitness variables for the authors: one based on the number of publications and the other based on the number of collaborators. This study reveals that, for Hep-Th, both the number of publications of an author and the number of her/his collaborators are able to provide a good agreement with real data, while, for Automatic Driving, we found a better performance of the model with the weights based on the number of publications. Probably this difference is due to the fact that, while, in the considered temporal window, the Physics of High Energies is quite an old subject in which different branches developed, Automatic Driving is a much younger research area. (Indeed, the observed values of *T* and *L*_*T*_, that is the number of publications and the number of keywords in the considered period, for the Automatic Driving are much smaller than the ones observed for Hep-Th in the same period. The indicator ${\bar{N}}_{T}$ also suggests that Automatic Driving is a younger research field than Hep-Th, since the observed value for the former is greater than the one for the latter.) The behavior of the considered on-line social network results well described with a different kind of weights. We examined the dataset of Instagram, with posts considered as actions and hashtags as features, and we observed that the less followers a user has the higher the number of old hashtags used. This could be related to the fact that less popular users tend to re-use many old hashtags in order to increase their visibility, while highly famous users do not feel the need of improving their popularity in this way and focus on few old hashtags. Indeed, this behavior seems to show a different role of the “on-line followership” relations respect to coauthorships: while collaborations incentive the usage of a high number of existing features, the number of followers takes to a limited usage of existing hashtags. Regarding this application, we also observed that, as in the considered collaboration networks, the relevance of an agent (with respect to the probability of transmitting her/his features) is well measured by her/his activity, that is the number of her/his actions.

## Supporting information

S1 Text Some asymptotic results for the model, estimation of the model parameters and data cleaning procedure. (PDF)
