# Supplementary material for: Collaboration and followership: A stochastic model for activities in social networks
Source: PLoS One. 2019 Oct 30;14(10):e0223768. doi: 10.1371/journal.pone.0223768 (PMC6821107; doi:10.1371/journal.pone.0223768)
Supplement: S1 Text — (PDF) [file pone.0223768.s001.pdf]

Collaboration and followership: a stochastic model for activities in social networks

Carolina Becatti, Irene Crimaldi, Fabio Saracco

A Supplemental file

We here collect the technical results and details that, for the sake of simplicity, have not been included in the main body of the paper. Specifically, in Subsection A.1 we describe the asymptotic behavior of the total number of features along time and we show some analytic findings regarding the asymptotic behavior of the mean number of edges in the actions-features bipartite network; in Subsection A.2 we provide some statistical tools in order to estimate the parameters of the model; finally, Subsection A.3 contains the cleaning procedure used for the IEEE and arXiv datasets.

A.1 Some asymptotic results for the model

We here illustrate some asymptotic properties of the model.

A.1.1 Asymptotic behavior of the total number of features

The random variable $L_t = \sum_{j=1}^{t} N_j$, that represents the total number of features present in the system at time-step t, has the following asymptotic behavior as $t \to +\infty$:

a) for $\beta = 0$, we have a logarithmic behavior of L_t, that is $L_t / \ln(t) \to \alpha$ almost surely;

b) for $\beta \in (0, 1]$, we obtain a power-law behavior, i.e. $L_t / t^{\beta} \to \alpha/\beta$ almost surely.

The proof of these two statements is exactly the same as in [1], since the weights do not affect L_t.

A.1.2 Asymptotic behavior of the mean number of edges in the actions-features network

We here analyze the asymptotic behavior, as $t \to +\infty$, of $\mu_e(t) = E[e(t)]$, where $e(t)$ is the total number of edges in the actions-features network at time-step t, that is the total number of ones in the matrix F until time-step t. A first remark is that we have

$$e(t) = \sum_{u=1}^{t} \sum_{k:\, T_k = u} d_k(t), \tag{1}$$

where we denote by T_k the arrival time-step of feature k and

$$d_k(t) = \sum_{j=1}^{t} F_{j,k} = 1 + \sum_{j=T_k+1}^{t} F_{j,k} \tag{2}$$

is the degree of feature k at time-step t. Hence, we can write

$$\begin{aligned} E[e(t)|T_k\ \forall k \,\text{with}\, T_k \le t] &= \sum_{u=1}^{t} \text{card}(k:\ T_k = u) E[d_k(t)|T_k = u] \\ &= \sum_{u=1}^{t} N_u E[d_k(t)|T_k = u], \end{aligned} \tag{3}$$

where we recall that N_u is Poi(λ_u)-distributed with $\lambda_u = \alpha/u^{1-\beta}$. In the following subsections, we go further with the computations in the two extreme cases $\delta = 1$ and $\delta = 0$ since the behavior for a general δ is a mixture of the two behaviors in the extreme cases. A graphical representation of the evolution of $\mu_e(t)$ in the considered cases is provided in Figure A.1 (the values are averaged over a sample of $R = 100$ simulations).

The case $\delta = 1$

In this case the inclusion probability of a feature k at time-step t simply is $P_t(k) = \frac{1}{2}$. Therefore, since (2), we have

$$E[d_k(t)|T_k = t_k] = 1 + \frac{t - t_k}{2} \sim t/2.$$

Hence, by (3) and the above approximation, we can approximate $\mu_e(t)$ by the quantity

$$\frac{t}{2} \sum_{u=1}^{t} \lambda_u = \frac{\alpha t}{2} \sum_{u=1}^{t} u^{\beta-1} \sim \frac{\alpha t^{1+\beta}}{2\beta}. \tag{4}$$

The case with $\delta = 0$ and the weights equal to a constant

Let us assume $\delta = 0$ and $W_{t,j,k}$ equal to a constant $w \in]0,1]$ for all t, j, k, so that the inclusion probability of a feature k at time-step t is

$$P_t(k) = \frac{d_k(t-1)}{t} w.$$

Let us set $\langle d_k(t) \rangle = E[d_k(t) | T_k = t_k]$ and observe that we have

$$\begin{aligned}
\langle d_k(t) \rangle =& 1 + w \sum_{\tau=t_k+1}^{t} \frac{\langle d_k(\tau-1) \rangle}{\tau} \\
=& 1 + w \left[\sum_{\tau=t_k+1}^{t-1} \frac{\langle d_k(\tau-1) \rangle}{\tau} + \frac{\langle d_k(t-1) \rangle}{t} \right] \\
=& 1 + w \sum_{\tau=t_k+1}^{t-1} \frac{\langle d_k(\tau-1) \rangle}{\tau} + \frac{w}{t} \left[1 + w \sum_{\tau=t_k+1}^{t-1} \frac{\langle d_k(\tau-1) \rangle}{\tau} \right] \\
=& \left(1 + \frac{w}{t}\right) \left[1 + w \sum_{\tau=t_k+1}^{t-1} \frac{\langle d_k(\tau-1) \rangle}{\tau} \right] \\
=& \cdots \\
=& \left(1 + \frac{w}{t}\right) \left(1 + \frac{w}{t-1}\right) \cdots \left(1 + \frac{w}{t_k+1}\right) \\
=& \frac{t_k!}{t!} \cdot (t+w) \cdot (t-1+w) \cdots (t_k+1+w) \\
=& \frac{t_k!}{t!} \cdot \frac{(t+w) \cdot (t-1+w) \cdots (t_k+1+w) \cdot (t_k+w) \cdots (w+1) \cdot w}{(t_k+w) \cdots (w+1) \cdot w}.
\end{aligned}$$

Using the properties of the Γ-function, we can write

$$\langle d_k(t) \rangle = \frac{t_k!}{t!} \frac{\Gamma(t+w+1)!}{\Gamma(t_k+w+1)!} = \frac{\Gamma(t_k+1)}{\Gamma(t+1)} \frac{\Gamma(t+w+1)!}{\Gamma(t_k+w+1)!} \sim \left(\frac{t}{t_k}\right)^w. \tag{5}$$

Therefore, by (3) and the above approximation, we can approximate $\mu_e(t)$ by the quantity

$$\sum_{u=1}^{t} \lambda_u \frac{t^w}{u^w} = \alpha t^w \sum_{u=1}^{t} u^{\beta-w-1} \sim \begin{cases} \alpha t^\beta \ln(t) & \text{if } w = \beta, \\ \dfrac{\alpha}{\beta - w}(t^\beta - t^w) \sim \dfrac{\alpha t^{\max\{w,\beta\}}}{|w-\beta|} & \text{if } w \neq \beta. \end{cases} \tag{6}$$

Remark: It is worthwhile to note that in the case of weights of the form $W_{t,j,k} = W_t$ for all t, j, k, where the random variables W_t take values in $[0,1]$, are identically distributed with mean value equal to μ_W, and each of them is independent of all the past until time-step $t-1$, we get for $\mu_e(t)$ the same asymptotic behavior as above, but with $w = \mu_W$.

The case with $\delta = 0$ and the weights depending only on k

Let us assume $\delta = 0$ and $W_{t,j,k} = W_k$ for all t, j, k, where the random variables W_k take values in $[0,1]$, are independent and identically distributed with probability density function ρ, and each of them independent of the arrival time-step T_k of the feature. Moreover, we focus on the case $\beta < 1$, that is more interesting then the case $\beta = 1$. In this case the inclusion probability is

$$P_t(k) = \frac{d_k(t-1)}{t} W_k.$$

Using the same computations done above, we get

$$E[d_k(t)|T_k = t_k,\, W_k] \sim \left(\frac{t}{t_k}\right)^{W_k}$$

and so we can approximate $E[d_k(t)|T_k = t_k]$ by $\int_0^1 \left(\frac{t}{t_k}\right)^w \rho(w)\, dw$. Hence, using (3), we can approximate $\mu_e(t)$ by

$$\sum_{u=1}^{t} \lambda_u \int_0^1 \left(\frac{t}{u}\right)^w \rho(w) dw = \int_0^1 t^w \sum_{u=1}^{t} \lambda_u u^{-w} \rho(w) dw =$$
$$\alpha \int_0^1 t^w \sum_{u=1}^{t} u^{-(w-\beta+1)} \rho(w) dw = \alpha t^\beta \int_0^1 \frac{t^{w-\beta} - 1}{w - \beta} \rho(w) dw. \qquad (7)$$

Therefore the asymptotic behavior of $\mu_e(t)$ depends on the asymptotic behavior of the above integral. In the sequel we analyze the case of the uniform distribution and the one of the "truncated" exponential distribution. To this purpose, we employ the Exponential integral

$$\mathrm{Ei}(y) = -\int_{-y}^{+\infty} \frac{e^{-x}}{x} dx = \int_{-\infty}^{y} \frac{e^v}{v} dv,$$

which has the property $\lim_{y \to +\infty} \frac{e^y}{y\mathrm{Ei}(y)} = 1$.

Example 1 (*Uniform distribution on* $[0,1]$)
If $\rho(w) = 1$, $\forall w \in [0,1]$ and equal to zero otherwise, we can compute the above integral and approximate $\mu_e(t)$ by

$$\alpha t^\beta \left\{ \int_{-\beta \ln(t)}^{(1-\beta)\ln(t)} \frac{e^v}{v} dv - \int_{-\beta}^{1-\beta} \frac{1}{v} dv \right\} = \alpha t^\beta \Big\{ \mathrm{Ei}[(1-\beta)\ln(t)] - \mathrm{Ei}[-\beta \ln(t)] + \ln\left(\frac{\beta}{1-\beta}\right) \Big\} \qquad (8)$$

Using the asymptotic properties of the Exponential integral, we find that the above quantity behaves for $t \to +\infty$ as

$$\frac{\alpha t}{(1-\beta)\ln(t)}.$$

Example 2 (*Exponential distribution on* $[0,1]$)
If $\rho(w) = e^{1-w}/(e-1)$ for $w \in [0,1]$ and equal to zero otherwise, the computation of the above integral leads to the approximation for $\mu_e(t)$ given by

$$\frac{\alpha e^{1-\beta}}{(e-1)} t^{\beta} \left\{ -\int_{-\beta}^{1-\beta} \frac{e^{-x}}{x} dx + \int_{-\beta(\ln(t)-1)}^{(1-\beta)(\ln(t)-1)} \frac{e^{v}}{v} dv \right\}$$
$$= \frac{\alpha e^{1-\beta}}{(e-1)} t^{\beta} \Big\{ \mathrm{Ei}[\beta] - \mathrm{Ei}[-(1-\beta)] + \mathrm{Ei}[(1-\beta)(\ln(t)-1)] - \mathrm{Ei}[-\beta(\ln(t)-1)] \Big\}. \tag{9}$$

Using the asymptotic properties of the Exponential integral, we find that the asymptotic behavior for $t \to +\infty$ of the above quantity is given by

$$\frac{\alpha t}{(e-1)(1-\beta)\ln(t)}.$$

A.2 Estimation of the model parameters

We here provide some statistical tools in order to estimate the parameters of the model: α, β and δ. If we observe a number of T actions, let $\{F_1 = f_1, F_2 = f_2, \ldots, F_T = f_T\}$ be the observed actions-features matrix rows and let $\{N_1 = n_1, N_2 = n_2, \ldots, N_T = n_T\}$ be the observed numbers of new features introduced by each of the observed actions.

The parameters α and β

The parameters α and β can be estimated using a maximum likelihood method, that is maximizing the probability to observe $\{N_1 = n_1, N_2 = n_2, \ldots, N_T = n_T\}$. Since all the random variables N_t are assumed independent and Poisson distributed with parameter α for $t = 1$ and $\lambda_t = \alpha/t^{1-\beta}$ for $t \geq 2$, we have

$$P(N_1 = n_1, \ldots, N_T = n_T) = \mathrm{Poi}(\alpha)\{n_1\} \prod_{t=2}^{T} \mathrm{Poi}\left(\lambda_t\right)\{n_t\}. \tag{10}$$

Hence, we choose as estimates the pair $(\widehat{\alpha}, \widehat{\beta})$ that maximizes the function (10), or equivalently its log-likelihood expression

$$\ln\left(\mathrm{Poi}(\alpha)\{n_1\}\right) + \sum_{t=2}^{T} \ln\left(\mathrm{Poi}\left(\lambda_t\right)\{n_t\}\right).$$

Remark: From the result stated in Subsection A.1.1, we get that $\ln(L_t)/\ln(t)$ is a strongly consistent estimator for β. Indeed:

a) if $\beta = 0$, then we have $L_t \overset{a.s.}{\sim} \alpha \ln(t)$ as $t \to +\infty$, so $\ln(L_t) \overset{a.s.}{\sim} \ln(\alpha) + \ln(\ln(t))$ and hence $\ln(L_t)/\ln(t) \overset{a.s.}{\to} 0 = \beta$;

b) if $\beta \in (0, 1]$, then we have $L_t \overset{a.s.}{\sim} (\alpha/\beta)t^{\beta}$ as $t \to +\infty$, so $\ln(L_t) \overset{a.s.}{\sim} \ln(\alpha/\beta) + \beta \ln(t)$, and hence $\ln(L_t)/\ln(t) \overset{a.s.}{\to} \beta$.

The parameter δ

An estimate for the parameter δ is obtained maximizing the probability to observe $\{F_1 = f_1, F_2 = f_2, \ldots, F_T = f_T\}$. More precisely, we have

$$P\left(F_1 = f_1, \ldots, F_T = f_T\right) = P\left(F_1 = f_1\right) \prod_{t=2}^{T} P\left(F_t = f_t | F_1, \ldots, F_{t-1}\right) =$$

$$P\left(N_1 = n_1\right) \prod_{t=2}^{T} P\left(F_{t,k} = f_{t,k} \text{ for } k = 1, \ldots, L_{t-1},\ N_t = n_t | F_1, \ldots, F_{t-1}\right) =$$

$$\mathrm{Poi}\left(\alpha\right)\{n_1\} \prod_{t=2}^{T} \mathrm{Poi}\left(\lambda_t\right)\{n_t\} \left\{ \prod_{k=1}^{L_{t-1}} P_t(k)^{f_{t,k}} \left(1 - P_t(k)\right)^{1-f_{t,k}} \right\},$$

where $P_t(k)$ is the inclusion probability defined in the main body of the paper. Since many terms in the previous equation do not depend on δ, the problem simplifies into the choice of the value of $\widehat{\delta}$ that maximizes the following function

$$\prod_{t=2}^{T} \prod_{k=1}^{L_{t-1}} P_t(k)^{f_{t,k}} \left(1 - P_t(k)\right)^{1-f_{t,k}} \tag{11}$$

or, equivalently, taking the logarithm,

$$\sum_{t=2}^{T} \sum_{k=1}^{L_{t-1}} f_{t,k} \ln\left(P_t(k)\right) + \left(1 - f_{t,k}\right) \ln\left(1 - P_t(k)\right). \tag{12}$$

A.3 Data cleaning procedure

For the arXiv and IEEE datasets, the data preparation procedure has been carried out using the Python package *NodeBox* [1], that allows to perform different grammar analyses on the English language. We use the library to categorize (as noun, adjective, adverb or verb) each word in all title's or abstract's sentences, with the final purpose of selecting nouns and adjectives only. Then, all selected words are modified substituting capital letters with lowercases and transforming all plurals into singulars, again using the *NodeBox* package. Finally, we also remove special words such as "study", "analysis" or "paper", that may often appear in the abstract text but are not relevant for the description of the topic and for the purpose of our analysis. Authors names are similarly treated. Indeed, from each name we replace capital letters with lowercases and we modify it by considering only the initial letter for each reported name and the entire surname. To make an example, names such as "Peter Kaste" or "P. Jacob" are respectively transformed into "p.kaste" and "p.jacob". One drawback of this kind of analysis is that authors with more than one names who reported all of them or just some in different publications cannot be distinguished. Indeed, in this situation they would appear as distinct. For example "A. N. Leznov", "A. Leznov" or "Andrey Leznov" may probably identify the same person who reported respectively two initials, one initial or the full name in different papers. However, with this transformation they appear as two distinct authors, since they are respectively represented by the abbreviations "a.n.leznov" and "a.leznov". Despite this fact, no further disambiguation is performed on the names, since it would be computationally very expensive and outside the scope of this research work [2].

References

[1] Irene Crimaldi, Michela Del Vicario, Greg Morrison, Walter Quattrociocchi, and Massimo Riccaboni. Modelling Networks with a Growing Feature-Structure. *Interdiscip. Inf. Sci.*, 23(2):127–144, 2017.

[2] Ronald Lai, Alexander D'Amour, Amy Yu, Ye Sun and Lee Fleming. Disambiguation and Co-authorship Networks of the U.S. Patent Inventor Database (1975 - 2010) *Research Policy*, **43**: 941–955 (2014).

[1] https://www.nodebox.net/code/index.php/Linguistics#loading_the_library

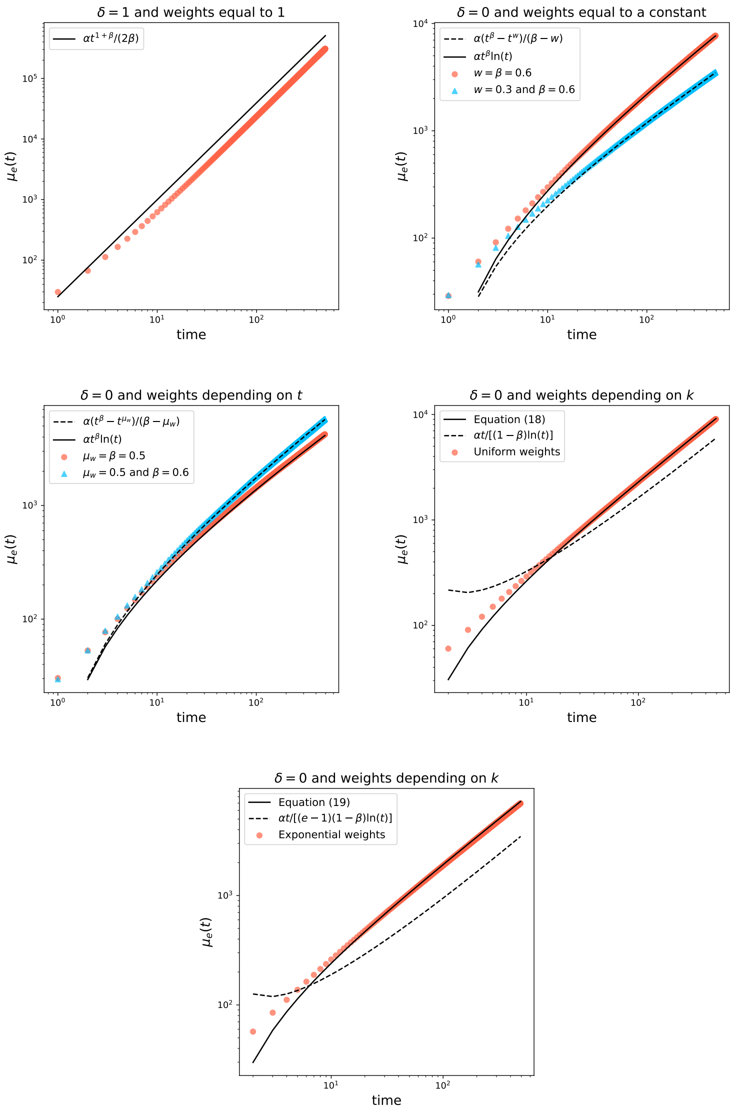

Figure A: Evolution of $\mu_e(t)$, i.e. the mean number of edges along time. From top-left to bottom-right we have the cases: $\delta = 1$ and weights equal to 1; $\delta = 0$ and weights equal to a constant w (the blue triangles represent the case with $w \neq \beta$, while the red dots show the case $w = \beta$); $\delta = 0$ and weights depending only on t with uniform distribution on $[0, 1]$ (the blue triangles show the case with the mean value $\mu_W \neq \beta$, while the red dots describe the case with $\mu_W = \beta$); $\delta = 0$ and weights depending only on k, considering the two different distributions of the provided examples (uniform and truncated exponential distribution) for the weights: the continuous lines refer to the values of the integrals (8) and (9), respectively, while the dashed lines show the final approximations. All simulations have been performed with $\alpha = 30$ and $\beta = 0.6$ (unless otherwise specified in the legend).
